# A Novel Compact Broadband Quasi-Twisted Branch Line Coupler Based on a Double-Layered Microstrip Line

**DOI:** 10.3390/mi15010142

**Published:** 2024-01-17

**Authors:** Fayyadh H. Ahmed, Rola Saad, Salam K. Khamas

**Affiliations:** Communications Research Group, Department of Electronic and Electrical Engineering, University of Sheffield, Sheffield S1 3JD, UK; r.saad@sheffield.ac.uk (R.S.); s.khamas@sheffield.ac.uk (S.K.K.)

**Keywords:** branch line coupler (BLC), microstrip double-layered TL (MDL-TL), phased array antenna, slow wave structure

## Abstract

A novel quasi-twisted miniaturized wideband branch line coupler (BLC) is proposed. The design is based on bisecting the conventional microstrip line BLC transversely and folding bisected sections on double-layered substrates with a common ground plane in between. The input and output terminals, each with a length of λ_g_/4, and the pair of quarter-wavelength horizontal parallel arms are converted into a Z-shaped meandered microstrip line in the designed structure. Conversely, the pair of quarter-wavelength vertical arms are halved into two lines and transformed into a periodically loaded slow-wave structure. The bisected parts of the BLC are placed on the opposite side of the doubled-layer substrate and connected through four vias passing through the common ground plane. This technique enabled a compact BLC size of 6.4 × 18 mm^2^, which corresponds to a surface area miniaturization by ~50% as compared to the classical BLC size of 10 × 23 mm^2^ at 6 GHz. Moreover, the attained relative bandwidth is 73.9% (4.6–10 GHz) for S11, S33, S21, and the phase difference between outputs (∠S21 − ∠S41). However, if a coupling parameter (S41) of up to −7.5 dB is considered, then the relative bandwidth reduces to 53.9% (4.6–10 GHz) for port 1 as the input. Similarly, for port 3 as the input, the obtained bandwidth is 75.8% (4.5–10 GHz) for S33, S11, S43, and the phase difference between outputs (∠S43 − ∠S23). Likewise, this bandwidth reduces to 56% (4.5–8 GHz) when a coupling parameter (S23) of up to −7.5 dB is considered. In contrast, the relative bandwidth for the ordinary BLC is 41% at the same resonant frequency. The circuit is constructed on a double-layered low-cost FR4 substrate with a relative permittivity of 4.3 and a loss tangent of 0.025. An isolation of −13 dB was realized in both S_13_ and S_31_ demonstrating an excellent performance. The transmission coefficients between input/output ports S_21_, S_41_, S_23_, and S_43_ are between −3.1 dB to −3.5 dB at a frequency of 6 GHz. Finally, the proposed BLC provides phase differences between output ports of 90.5° and 94.8° at a frequency of 6 GHz when the input ports 1 and 3 are excited, respectively. The presented design offers the potential of being utilized as a unit cell for building a Butler matrix (BM) for sub-6 GHz 5G beamforming networks.

## 1. Introduction

5G technology offers high channel capacity, a high data rate, and channel aggregation with low latency over MIMO fading environments. On the other hand, 5G components need to be compact to be incorporated into modern portable devices, such as smartphones and tablets, which tend to be slim and lightweight, while also requiring high processing capabilities [1].

**Figure 1:**
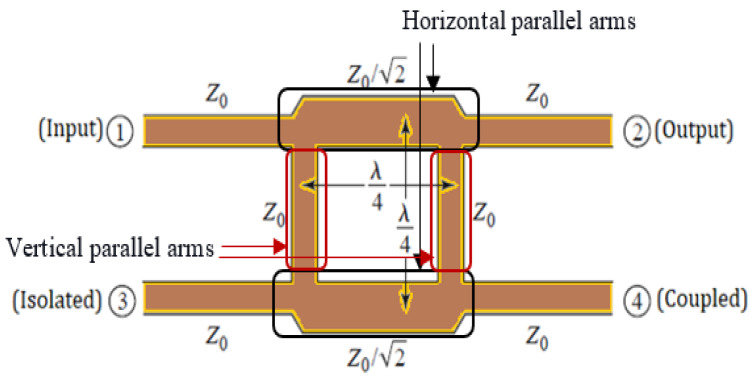
Geometry of a branch-line coupler.

Beam scanning antennas play a key role in 5G communication systems to attain the desired outputs. The use of beamforming feeding networks (BFNs) is an essential technique to obtain high directivity in a particular direction and improve the connection quality as well as coverage of 5G systems [2]. The function of BFNs is to adjust the phase and amplitude of feeding signals for phased array antenna systems [3]. The Butler matrix (BM) is one of the most common BFNs of 5G systems owing to distinctive features such as simplicity, lower cost, and an easy fabrication process. In addition, the BM does not need external biasing in its operation. Further, the BM operates as a reciprocal feeding system for transmitting and receiving signals in phased array antenna systems. The building block of the BM is the BLC, which can be utilized as a modulator, mixer, and phase shifter as well as its basic function as part of a feeding network for phased array antenna systems [2]. Therefore, this work focuses on the design of a compact BLC with an enhanced bandwidth. As illustrated in Figure 1, a BLC consists of four transmission lines grouped into two pairs, with each pair consisting of parallel horizontal and vertical lines. The characteristic impedance of the horizontal lines is Z_o_/√2, while for the vertical counterparts, it is Z_o_, where Z_o_ represents the characteristic impedance of the microstrip transmission line (MSTL). Each of the four transmission lines (TL) has a length of λ_g_/4, where λ_g_ is the guided wavelength. Thereby, the dimensions of the BLC are primarily based on the operating frequency, and thus in low frequencies, the BLC extends over a large area of the host device board and hence leads to an increased size [4]. Another inherent issue with a conventional BLC is the limited bandwidth characteristics of no more than 50% that restrict their applications and require a large multi-sections circuit to gain wideband characteristics, which in turn increase the circuit’s area [5].

On the other hand, in high-speed systems, microstrip lines find applications in transmitting signal pulses rather than analog microwave signals. These systems encompass various domains, including very high-speed computer logic (operating at GHz clock rates), high bit-rate digital communications, high-speed samplers for oscilloscopes or time-domain reflectometers, and radars [6]. One crucial characteristic of microstrip lines in these scenarios is propagation delay, which depends on the effective dielectric constant. However, there are situations where it becomes necessary to extend this delay. One approach to achieve this extension in effective propagation delay is by constructing a slow-wave transmission line. This can be accomplished, for instance, by introducing capacitive loading at intervals along the microstrip. In such cases, the delay is influenced by these capacitances, leading to a reduction in *ν*p (velocity of propagation) and effectively slowing down the pulse when the capacitance (C) is increased [6].

Many efforts have been reported that address the above limitations. For example, the concept of coupled line unit cells was introduced in [4] to create a dual composite right/left-handed (D-CR/LH) unit cell, which results in a design that has a miniaturized area of ~52% of that of a conventional BLC at 1.8 GHz with a relative bandwidth of 18%. T-shaped slots and open stubs were employed in the horizontal and vertical arms of the BLC, resulting in a 30% bandwidth improvement and a 12.3% size reduction [5].

In another study, a double-layer board with slow-wave microstrip transmission lines and blind vias was used to achieve a 43% size reduction compared to a conventional design at the same resonant frequency. However, the increased number of vias led to higher insertion losses and design complexity [7]. A Koch fractal-shape BLC of various iterations was suggested in [8], where the sample was designed to operate at 2.4 GHz and offered a size reduction of ~81% in combination with a relative bandwidth of 33%.

In [9], a compact artificial transmission line was proposed for compact microwave components. The transmission line combines resonant-type composite right/left-handed transmission lines (CRLH TLs) with fractal geometry. Two sets of planar CRLH cell structures were provided: one based on a cascaded complementary single split ring resonator (CCSSRR), and the other based on complementary split ring resonators (CSRRs). A dual-band bandpass filter (BPF) and a monoband branch line coupler were designed based on the suggested artificial line.

The effectiveness of integrating CRLH TL and fractal geometry for designing compact broadband microwave devices was confirmed in [10]. In this study, a proposal was made for a compact balun with improved bandwidth, utilizing a completely artificial fractal-shaped composite right/left-handed transmission line (CRLHTL). Chip components were employed for the left-handed contribution, and fractal microstrip lines were utilized for the right-handed part, focusing on miniaturization. This innovative technology provided an extra degree of flexibility in crafting compact devices and demonstrates superiority over alternative methods.

In [11], open-ended stubs and transmission line meandering with a stepped impedance approach was proposed with a size reduction of ~61% and 50% compared to a conventional BLC, respectively. However, the narrow bandwidth of ~130 MHz represents a key limitation. A flexible coupler using a Teslin paper substrate was reported [12]. It replaced the conventional quarter-wavelength transmission lines with a collective of shunt open-stubs, series transmission lines, and meandered lines, resulting in a compact design with a surface area of 0.04 $\lambda_{g}^{2}$ and a 68% fractional bandwidth. Using a dual microstrip transmission line, the BLC size was reduced by 32% with a fractional bandwidth of 60% [13]. However, this approach had poor return losses over the operating bandwidth. To improve matching, T-shaped transmission lines were used, reducing fractional bandwidth and size to 50% and 44%, respectively. A compact BLC class introduced a prototype using open-circuited stubs to replace traditional quarter-wavelength transmission lines [14], resulting in a ~55.6% size reduction and achieving 11% and 50% fractional bandwidths for narrowband and wideband modes of operation, respectively.

In [15], a new configuration, BLC, is presented. The design applies two types of trapezoid-shaped resonators on the arms of the BLC to configure a wideband branch-line coupler. The proposed design achieved a size reduction of 79% compared to conventional couplers. In addition, it offers a fractional bandwidth of 22.2%. Ref. [16] used artificial transmission lines (ATL) for miniaturization. They replaced conventional transmission lines with right-handed transmission lines (RHTL) and constructed the branch-line coupler sides using cascaded T-Net RHTLs instead of quarter-wavelength transmission lines. This design achieved a 50% size reduction compared to the conventional BLC and a 33.3% fractional bandwidth (2.0–2.8 GHz). A simple method was used to improve bandwidth in [17]. By adding a single transmission line element to a conventional coupler, they increased bandwidth by approximately 25%. However, the proposed structure is larger at 25.7 × 22.8 mm^2^ compared to the conventional coupler’s 21.5 × 20.7 mm^2^.

Triangular and trapezoidal resonators were added to the coupler for miniaturization and harmonic suppression [18]. The design achieved an 84% size reduction and wide harmonic suppression. However, it has a complex structure with a low-frequency band around 200 MHz, representing a 26% fractional bandwidth (FBW). A bandpass filter operating in three frequency bands utilized a dual-layer structure with distinct dielectric constants, as described in [19]. The dual-layer design was employed to diminish the overall size and enhance the isolation between the passbands. Consequently, the suggested configuration offers benefits such as a nearly 50% reduction in physical size and the alleviation of design constraints by utilizing the two substrates within a unified structure.

The majority of the aforementioned prototypes were based on composite right/left-handed structures to create branch lines, which might result in unfavorable characteristics that are associated with miniaturization such as shallow return losses for input ports, poorly isolated ports, and narrow bandwidths in some cases [3,4,6,8]. In addition, the structures of right/left-handed transmission lines probably increase the structure’s complexity, which results in a challenging practical realization despite the overall size reduction. 

In this study, a quasi-twisted shape branch line coupler is proposed, which is the longitudinal bisection of a conventional BLC into two sections and twisting each over the other. The structure is designed based on the microstrip double-layered TL (MDL-TL). The input/output transmission lines and horizontal arms of the BLC are built based on a Z-shape meandered section with round blend edges, while the λ_g_/4 vertical arms of the BLC are adopted for the slow wave structure. The MDL-TLs are placed on two layers and connected using four conductive vias. A common ground plane is placed between the layers of the MDL-TLs, which incorporate circular slots around the vias to avoid shorting them to the common ground plane. The described configuration reduced the size of the conventional BLC by 49.9% and improved the relative bandwidth to 75.8%. The novel design is modelled and simulated using a computer simulation technology (CST) microwave studio and then fabricated and tested on a low-cost FR-4 substrate material demonstrating promising S-parameter results. 

This paper is organized as follows: Section 2 explains the theoretical analysis and design procedures of developing a wideband MDL-TL and compares the achieved performance with that of a conventional microstrip line; Section 3 presents the analysis and design of a branch line coupler based on MDL-TL; finally, Section 4 presents the simulated and measured results demonstrating the novel BLC performance.

## 2. Theory

### 2.1. Selection of the Classical Microstrip Line Dimensions

The microstrip transmission line (MSTL) represents the building block of any passive and active microwave device due to a number of advantages, such as the easy fabrication process as well as the availability of numerous miniaturization approaches [20]. 

The width (*W*) of a microstrip line, situated on a thin grounded dielectric substrate with a height (*h*) and a dielectric constant (*ε_r_*), can be calculated for a specific characteristic impedance (*Z*_0_) as described in [20].

The microstrip physical length, *l*, that is required to generate a phase shift (delay) of *θ* can be determined as [20]:

(1) $$\theta =\beta l=\sqrt{\varepsilon_{e}}\;k_{o}l$$

where *f* and *c* are the frequency and speed of light, respectively, and $k_{o}=\frac{2\pi f}{c},$ $\varepsilon_{e}$ is the effective dielectric constant, which is in the range of $1<\varepsilon_{e}<\varepsilon_{r}$.

### 2.2. The Impact of Right-Angled Bend on the Performance of the Microstrip Line

**Figure 2:**
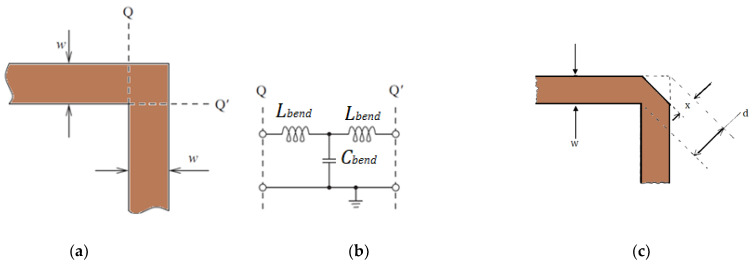
(**a**) Part of bend microstrip line, (**b**) Equivalent circuit, (**c**) Chamfered right-angled bend microstrip line.

Complex microwave circuits usually comprise bend microstrip transmission lines, and quite often the width of the line does not change across the bend. Figure 2 illustrates a right-angled bend MSTL with its equivalent circuit. The bend MSTL generates capacitance, *C_bend_*, and inductance, *L_bend_*, which result in the gathering of additional charge at the line corner in particular at the outer edge of the bend area, whereas inductance arises due to the disruption of current flow [6]. Closed formulas for the capacitance and inductance of the bend are determined in [6].

Generally, performance and bandwidth enhancement of a microstrip line-based circuit is realized by compensating bend discontinuities. This is usually implemented by chamfering or rounding the corners, which leads to the minimizing of reactance. The percentage chamfer, *M*, is given by (*x/d*) × 100%, where *x/d* is chamfered to bend at a diagonal ratio at the bending corner as in Figure 2c and can be determined using [6]:

(2) $$M=52+65e^{\left(-1.35W/h\right)}$$

which is valid for $W/h\geq 0.25\;\mathrm{and}\;\varepsilon_{r}\leq 25$.

In this work, the MDL-TL is used as a unit cell to build the BLC. This line is bent over a horizontal *xy* plane and vertical *xz* or *yz* planes when folded around double-layer substrates. The normal even-and odd-mode theory can be used to analyze the port parameters of a pair of quasi-twisted MDL-TLs. 

**Figure 3:**
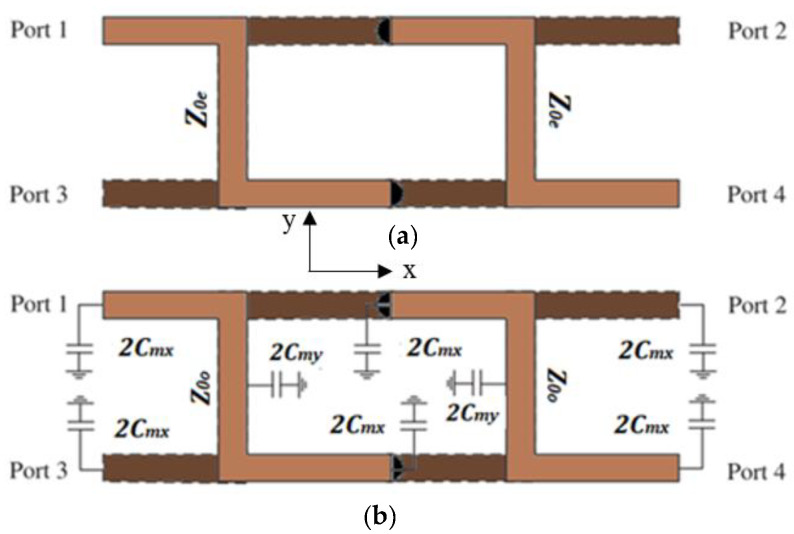
Double-layered MSTL equivalent model: (**a**) even mode; (**b**) odd mode.

The schematics of the meandered Z-shaped MSTL in even and odd modes are demonstrated in Figure 3a,b. The *C_mx_* and *C_my_* are the coupling capacitances in the *x* and *y* directions, respectively, factor 2 arises due to double-layered structure around the common ground plane, and *C_my_* is much stronger than *C_mx_*. Therefore, it can be neglected [21]. The difference in the characteristic impedance between the even and odd modes can be negligible since the coupling between top and bottom MSTL is very small, and they are given as in [21]:

(3) $$Z_{0e}=Z_{0}\sqrt{\frac{1+C}{1-C}},\;Z_{0o}=Z_{0}\sqrt{\frac{1-C}{1+C}}$$

where *C* is the coupling factor.

### 2.3. Study of the Performance of the MDL-TL

**Figure 4:**
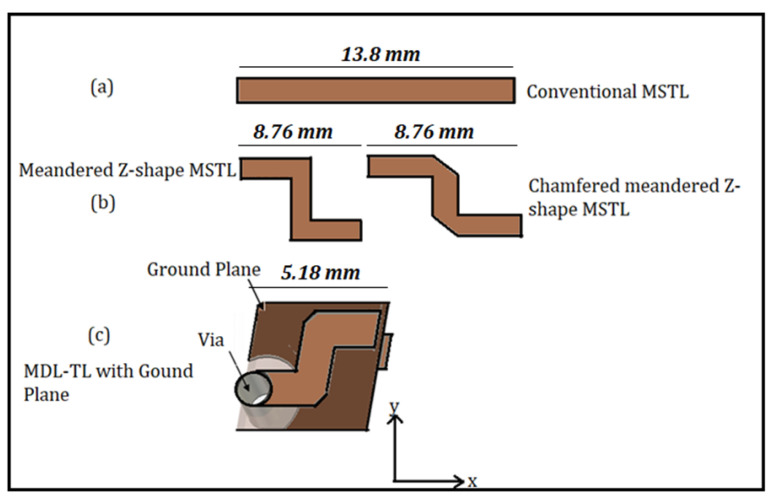
Various MSTL configurations: (**a**) single-layer straight MSTL; (**b**) single-layer meandered MSTL; (**c**) double-layer meandered MSTL.

This section aims to assess the effectiveness of the MDL-TL structure compared to a conventional microstrip transmission line by examining its S-parameters and comparing them with conventional ones. In order to create an MDL-TL structure from a conventional MSTL, four stages of modification are undertaken and can be observed in Figure 4. These stages include the one-layered conventional MSTL, the one-layered meandered MSTL without and with chamfering, and the double-layered with chamfering (MDL-TL). The effect of meandering of the structure and varying the line length over the *xy* plane and *x*-*z* plane was conducted on an FR-4 substrate with a dielectric permittivity of 4.4, a loss tangent of 0.025, and a substrate thickness of 0.8 mm at 6 GHz. This investigation includes a comparison of the key parameters of the four-line configurations: the reflection coefficient (S_11_), transmission coefficient (S_21_), and phase of S_21_ (or output phase).

In this study, a specific section of a λ_g_/2 length transmission line at 6 GHz, which corresponds to a physical length of 13.8 mm, was chosen. The width of the line was set at 1.52 mm to maintain a characteristic impedance of 50 Ω. The transmission line was transformed into a meandered Z-shape and then into an MDL-TL configuration using vias with diameters equal to the width of the microstrip line. The meandered Z-shape had a length of 8.76 mm, while the MDL-TL configuration had a length of 5.18 mm, as illustrated in Figure 4.

**Figure 5:**
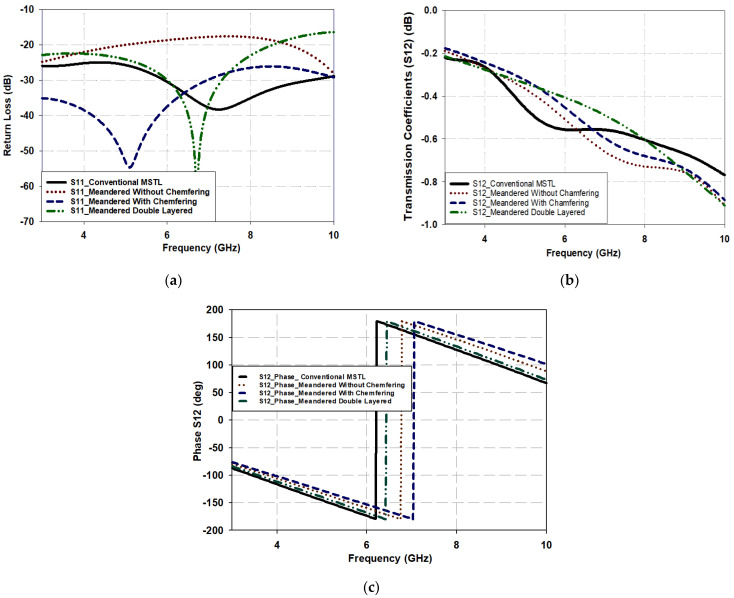
Performance of different MSTL configurations: (**a**) S11 (**b**) S12; (**c**) phase of S12 (output phase).

Figure 5a demonstrates the reflection coefficient, S_11_, of the four types of lines, where it can be noted how meandering the line without applying the chamfering technique deteriorates the impedance matching at the input ports by increasing S_11_ from −30 dB to −18 dB at a target frequency of 6 GHz. This is attributed to the emergence or occurrence of bending parameters like *C__bend_* and *L__bend_*, as explained earlier. However, it is also clear from Figure 5a how chamfering the corners provides discontinuity substitution and improves the matching again by reducing S_11_ to ~−38 dB. It can also be noticed that the S_11_ of the MDL-TL is as well matched as the conventional MSTL at 6 GHz. Moreover, the proposed MDL-TL structure offers as good matching as a conventional line as it has an almost equal and lower S_11_ than a conventional line, especially at a target frequency of around 6 GHz.

Figure 5b demonstrates the transmission coefficients of all line configurations, where it can be noted that adjusting the MSTL with the meandering, chamfering, etc. of a one-layered MSTL does not have much of an effect on the transmission coefficient since S_21_ is within −0.45 dB to −0.55 dB at 6 GHz. Furthermore, the S_21_ of the double-layered MSTL is higher by about 0.075 dB in the frequency range of 6 GHz to 7 GHz, which confirms a good performance compared to a conventional MSTL. On the other hand, the output phases of all configurations have approximately the same phase delay of 180° since the MSTL is modeled with an electrical length of λ_g_/2 at 6 GHz, as presented in Figure 5c. It should be noted that the same results were obtained when port 2 is considered as the input port, and they have been omitted for brevity. The achieved results confirm that the proposed configuration can be utilized as a unit cell for compact-size structures with improved performance.

## 3. Design of Branch Line Coupler

### 3.1. Conventional Branch Line Coupler

**Figure 6:**
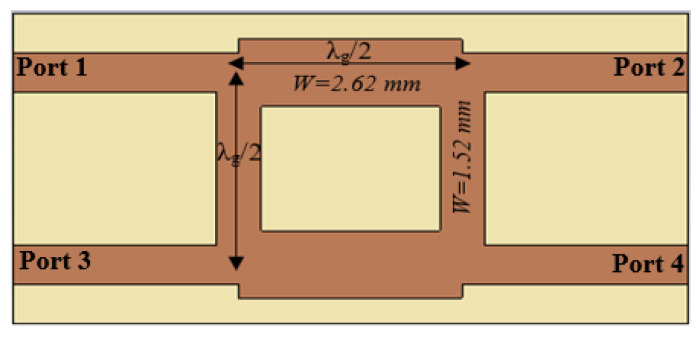
Structure of the conventional branch coupler operating at 6 GHz.

Figure 6 shows a 10 mm × 23 mm conventional BLC designed at 6 GHz on an FR4 substrate with a thickness of 0.8 mm and dielectric constant (*ε_r_*) of 4.4 with a loss tangent of 0.025, which are the same substrate specifications as the proposed BLC for an effective comparison. The 50 Ω microstrip line sections for ports 1, 2, 3, and 4 are designed with a width of 1.52 mm, and the λ_g_/4 sections of the coupler with an impedance of Z_0_/√2 (35.35 Ω) are of a microstrip line with a width of 2.62 mm, as shown in Figure 6. 

**Figure 7:**
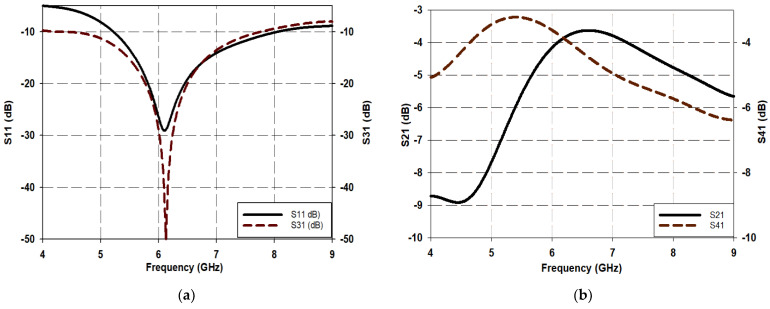
Scattering parameters of conventional BLC: (**a**) reflection coefficient (S_11_) and isolation coefficient (S_31_); (**b**) transmission coefficient (S_21_) and coupling coefficient (S_41_).

Figure 7 shows the simulated scattering parameters for the conventional branch line coupler operating at 6 GHz. The input reflection coefficient (S_11_) and the isolation coefficient between input ports, S_31_, are presented in Figure 7a, demonstrating a −30 dB excellent match for the conventional BLC at the frequency of interest, 6 GHz, alongside a relative impedance bandwidth of 42.2% (5.214–8 GHz), in addition to a perfect isolation of −50 dB. On the other hand, Figure 7b illustrates the transmission coefficient (S_21_) and coupling coefficient (S_41_), where it can be seen that the power is divided equally between the output ports (2 and 4) at 6.18 GHz with a value of −3.8 dB. However, the delivered power declines in both output ports (2, and 4) as the frequency increases, reaching −5.6 dB at the transmission port (port 2) and −6.38 dB at the coupled port (port 4), as shown in Figure 7b.

### 3.2. Proposed Quasi-Twisted Branch Line Coupler Structure

**Figure 8:**
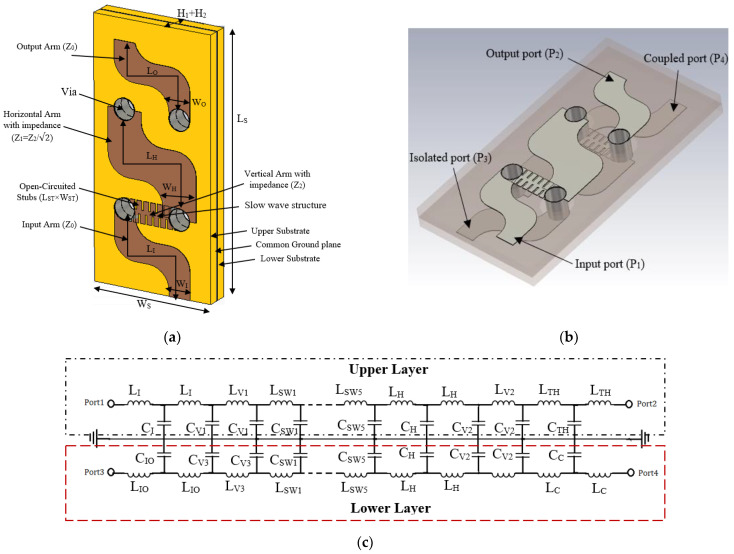
Proposed quasi-twisted branch line coupler: (**a**) perspective view; (**b**) frame mode view; (**c**) LC equivalent circuit.

A branch line coupler incorporating an MDL-TL topology was designed on a double-layered 0.8-mm FR-4 substrate, as shown in Figure 8. The horizontal λ_g_/4 dimensions and input/output ports’ ends of the conventional branch line coupler of Figure 1 were transformed to an MDL-TL-based Z-shape, while the vertical λ_g_/4 dimensions were converted to a periodically loaded open-stub configuration (slow wave structure) to achieve a compactness in the structure. Figure 8a presents a perspective of the proposed design. The preliminary dimensions of the suggested BLC were selected based on design equations of [20] for the conventional MSTL’s width and length, respectively. These dimensions were then slightly optimized using CST. The bending of the meandered Z-shape of the MDL-TL generates a certain reactive component that negatively affects the transmission line performance, such as reflection and transmission coefficients. Therefore, to compensate for this reactance, a 1.5 mm radius round-chamfering was implemented at the bending of the MDL-TL. A summary of the designed BLC specifications is presented in Table 1. The proposed branch line coupler configuration has a quasi-twisted structure as illustrated in Figure 8b. 

The approximate equivalent LC circuit of the proposed coupler is drawn as shown in Figure 8c. The circle was drawn for the proposed design based on mapping several equivalent circles to various components. These include the equivalent circle for the radial bend, representing the meandered transmission part [6,19], the equivalent circuit for the upper and lower slow-wave structures [6] as well as the equivalent circuit for four vias [22]. The complete equivalent circuit connects all the equivalent circuits together for the two layers of the proposed model.

In the Figure 8c circuit, L_I_, C_I_, L_TH_, C_TH_, L_IO_, C_IO_, and L_C_, C_C_ represent the inductances and capacitances of the equivalent circuit of the input through isolated and coupled ends, respectively. Meanwhile, L_SW1_ to L_SW5_ and C_SW1_ to C_SW5_ represent the equivalent circuit inductances and capacitances of the five sections of the slow wave structures on both the upper and lower substrates, respectively. Finally, L_V1_, C_V1_, L_V2_, C_V2_, L_V3_, C_V3_, L_V4_, and C_V4_ represent the equivalent circuit inductances and capacitances of the four vias of the proposed design.

The proposed design concept is inspired by twisted cable shapes, aiming to substantially reduce the blank space occupied by traditional BLC circuitry. This was accomplished by dividing the conventional BLC horizontally and interweaving the upper and lower segments in a twisted fashion, utilizing a Z-shaped meandering technique with traditional MSTL components. The design procedure of the suggested BLC in Figure 8 involves two key steps as follows.

Step 1: As shown in Figure 8, the characteristic impedance of the input, output, coupled, and isolated ports was selected to be Z_0_ = 50 Ω. Also, using the equation in [20] the relevant lines’ λ_g_/4 MSTL (the four input/output ports), shown in Figure 8, are designed with a width of 1.53 mm. On the other hand, the width of the horizontal characteristic’s impedance of Z_1_ = Z_0_/√2, is calculated as 2.63 mm and further optimized to 2.42 mm to realize the optimal s-parameter’s performance. The vertical λ_g_/4 length arms with an impedance of Z_2_ = Z_0_ are designed to occupy an optimal small area. The internal and external corners of all the Z-shaped sections are round-chamfered with a radius of 1.5 mm to improve the reflection and transmission coefficients, as described in Section 2. All inputs and outputs ending with a length of λ_g_/4 are converted to a meandered Z-shape. This is undertaken to eliminate the blank space occupied by these ends and achieve the most compact BLC possible. The length, *L_o_*, of the output port (*P*_2_) is less than λ_g_/4, which in turn is shorter than the *L_c_* length of the coupled port (*P*_4_), as shown in Figure 8b. These modifications in the lengths are necessary to tune the differences between the output ports’ phases to ~90°. The proposed structure, demonstrated in Figure 8, provides a novel BLC configuration, which is significantly miniaturized by ~50% as compared to a conventional BLC operating in the same frequency of 6 GHz.

**Figure 9:**
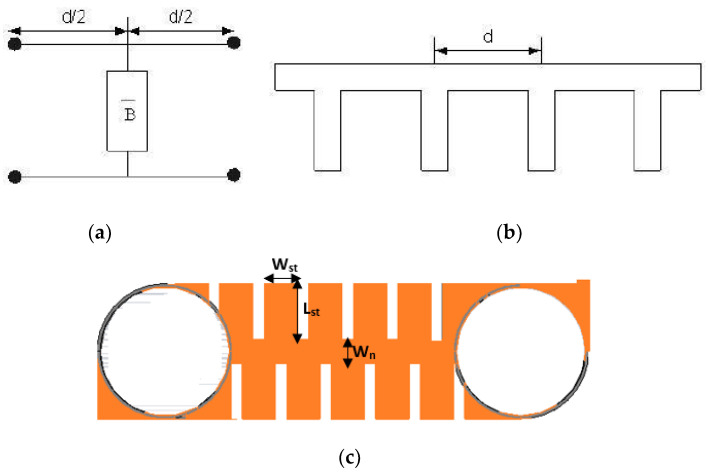
(**a**) A single periodic section circuit diagram of periodically loaded transmission line; (**b**) schematic diagram of periodically loaded line with open stub used as loaded capacitance; and (**c**) the proposed slow wave structure.

Step 2: The idea behind a slow wave structure involves the incorporation of shunt capacitors at regular intervals along the length of the transmission line, as illustrated in Figure 9a,b [23]. This technique results in decreasing both the characteristic impedance and phase velocity, as can also be modelled from Equations (7) and (8) below [23].

(4) $$Z_{0\_Loaded}=\sqrt{\frac{L}{C+\frac{C_{p}}{d}}}$$

(5) $$V_{p-Loaded}=\frac{1}{\sqrt{L\left(C+\frac{C_{p}}{d}\right)}}$$

where *C_p_* represents the periodically added capacitor at a distance, *d*, along the transmission line. *Z*_0*_loaded*_ and *Z*_0_ denote the characteristic impedance of the loaded and unloaded lines, respectively. On the other hand, reducing the phase velocity facilitates the achievement of an effectively longer electrical length by utilizing a physically shorter length. 

In the proposed design, a periodically loaded slow wave structure, as shown in Figure 9c, is adopted to design a compact λ_g_/4 line with an enhanced bandwidth to accommodate the space limitation introduced due to the folding of the BLC halves and the use of a double-layered Z-shaped meandering technique to realize compactness. Additionally, to obtain a line with a specific characteristic impedance, the loading section (*W_n_*) should possess a higher characteristic impedance, such that its characteristic impedance is decreased to the desired characteristic impedance, usually of 50 Ω impedance, after loading.

The relation between the physical length, *l_p_*, and the electric length, *l_e_*, of the periodically loaded stub TL is given as [23]:

(6) $$l_{e}=l_{p}\left(\frac{\omega_{0}}{V_{p-Loaded}}\right)$$

The added capacitance *C_p_*, in terms of the known parameters of the loaded and unloaded TL, is given as [24]:

(7) $$C_{p}=\frac{\phi }{N\omega_{0}}\left(\frac{Z_{o}^{2}-Z_{o\_loaded}^{2}}{Z_{o}^{2}Z_{o\_loaded}}\right)$$

where $\omega_{0}$ is the angular frequency, and *N* is an integer that refers to the stub section’s number.

On the other hand, open stubs with a length of multiples of a quarter wavelength were added in parallel to one pair of branch-line coupler sides, operating as a parallel stub transformer as reported in [25]. Therefore, the characteristic impedance of the added stubs attenuates the maxima in return loss characteristics (S_11_) when the frequency deviates, which, in turn, broadens the bandwidth. 

In the proposed configuration, shown in Figure 8, the slow wave structure is accomplished by inserting alternate slots around the conventionally straight MSTL with respective length and width of 2.62 mm and 1.52 mm, which creates rectangular stubs around both sides of the line as shown in Figure 9c. To create rectangular stubs around the MSTL, slots were placed in an alternate configuration, resulting in stub dimensions of W_st_ × L_st_ = 0.234 mm^2^ on both sides of the line as depicted in Figure 9c. The addition of these slots transforms the 50 Ω MSTL to a narrow MSTL with a width of W_n_ = 0.271 mm that provides a characteristic impedance of 105.9 Ω, which compensates for the shunt impedances of the periodic stubs and results in an overall characteristic impedance that is close to 50 Ω.

## 4. Fabrication and Measurements

**Figure 10:**
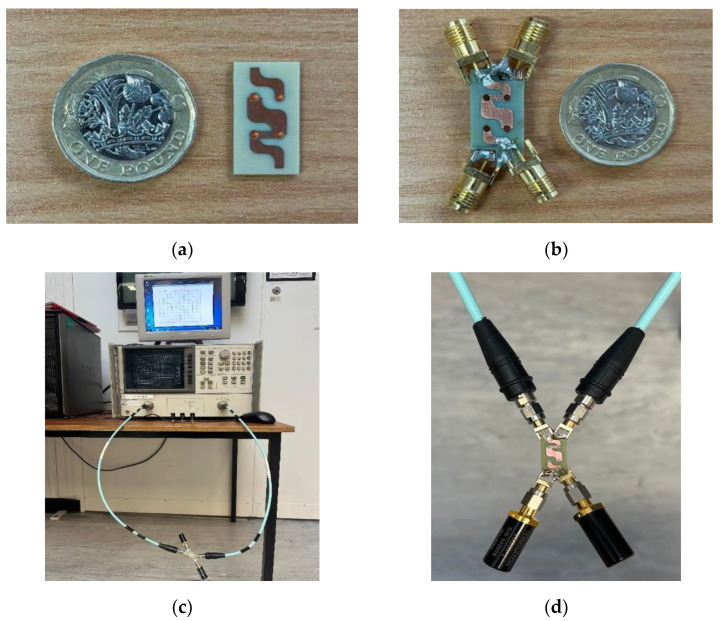
(**a**) BLC prototype, (**b**) BLC prototype with SMA connectors, (**c**) measurement setup system, and (**d**) termination of un-fed ports.

The proposed novel miniaturized BLC, shown in Figure 10, was fabricated on an FR4 substrate with a dielectric constant of 4.4 and a thickness of 0.8 mm, where a thin ground plane of 70 µm thickness was inserted between the two FR4 substrates, forming a sandwich-like structure. Both substrates were truncated at their corners using cross-sectional areas of 2 × 5 mm^2^ each to expose the ground plane and enable the SMA connector to be easily connected, as illustrated in Figure 10b. 

The fabricated novel BLC was measured using an HP 8720B vector network analyzer (VNA) from Test Equipment Center, Inc., Gainesville, FL, USA, as shown in Figure 10c. The reflection coefficient and isolation coefficient between input ports 1 and 3 as well as the transmission and coupling coefficients were measured by connecting the relevant ports to the VNA, while the remaining ports were terminated by a 50 Ω load to prevent additional mismatching and increase the measurements’ reliability, as illustrated in Figure 10d.

As per the design specifications of the proposed BLC, the required phase difference between the output signals is 90°. This phase difference can be verified by measuring the phases of the transmission coefficients, S_21_ and S_43_, and coupling coefficients, S_41_ and S_23_, at the output ports 2 and 4. Once these measurements are carried out, the phase difference can be determined as follows:

(8) $$\varphi =\{\begin{array}{c} ∠S_{21}-∠S_{41}\;\;\;\;\;\;\;\mathrm{for input from port}\;1\\ ∠S_{43}-∠S_{23}\;\;\;\;\;\;\mathrm{for input from port}\;3 \end{array}$$

**Figure 11:**
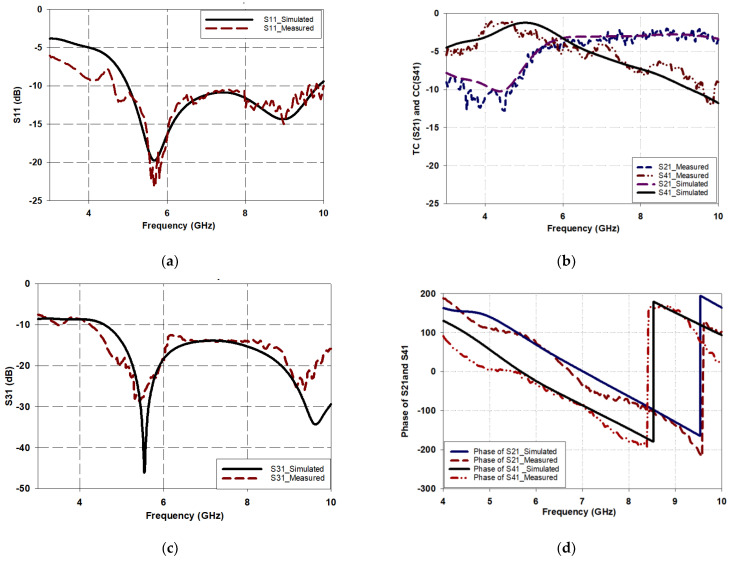
Four-port S-parameters for the proposed BLC when port 1 is excited: (**a**) S11, (**b**) S21 and S41 (**c**) S31, and (**d**) phase of S21 and S41.

The performance of the proposed BLC is evaluated by observing the four-port S-parameters’ magnitudes and phase differences as shown in Figure 11. The four principal scattering parameters considered in the analysis are as follows: the reflection coefficient (RC) S_11_, transmission coefficient (TC) S_21_, isolation coefficient (IC) S_31_, and coupling coefficient (CC) S_41_, when port 1 is excited as the input port. On the other hand, when port 3 is excited, the required scattering parameters, S_13_, S_23_, S_33_, and S_43_, are considered. Ports 1 and 3 were chosen as they are located on opposite sides of the coupler and are designated as input ports. In addition, from Figure 11a, it is evident that a good agreement was accomplished between the simulated and measured reflection coefficients. For example, the measured −10 dB S_11_ bandwidth extends from 4.6 GHz to 10 GHz, which corresponds to a relative bandwidth of 73.9% compared to a typical bandwidth of ~40% from an identical traditional branch line coupler. As a result, the proposed configuration offers a substantial bandwidth enhancement.

The transmission and coupling coefficients, S_21_ and S_41_, respectively, are presented in Figure 11b with good agreement between measurements and simulations. From these results, it can be observed that both the transmission and coupling coefficients are −3.9 dB at the desired frequency of 6 GHz, which is close to the ideal value of −3 dB. Notably, S_21_ remains higher than −3.5 dB for the entire operating band ranging from 5.8 GHz to 10 GHz. However, the coupling coefficient, S_41_, gradually degrades with increasing frequency, possibly due to vias and substrate losses since the input port and coupled ports are located on opposite sides of different substrates. Despite this degradation, the power delivered to port 4 remains greater than −7.5 dB up to a frequency of 8 GHz.

Furthermore, the isolation coefficient between the input ports, S_31_, as shown in Figure 11c demonstrates a magnitude of less than −13 dB throughout the operating band, signifying a good isolation between the input ports. This ensures that the proposed BLC meets the necessary design specifications for optimal performance.

Finally, Figure 11d presents the difference between the phases at the output ports 2 and 4 for input excitations from port 1. A 90° phase difference is required between the output signals. At a design frequency of 6 GHz, the phase difference between the output ports for inputs from port 1 is (∠S_21_ − ∠S_41_ = 90.50°), which satisfies the required phase difference for typical BLC design specifications. In addition, the phase difference error (PDE) is 0.5° for the designed frequency of 6 GHz, which is marginal.

**Figure 12:**
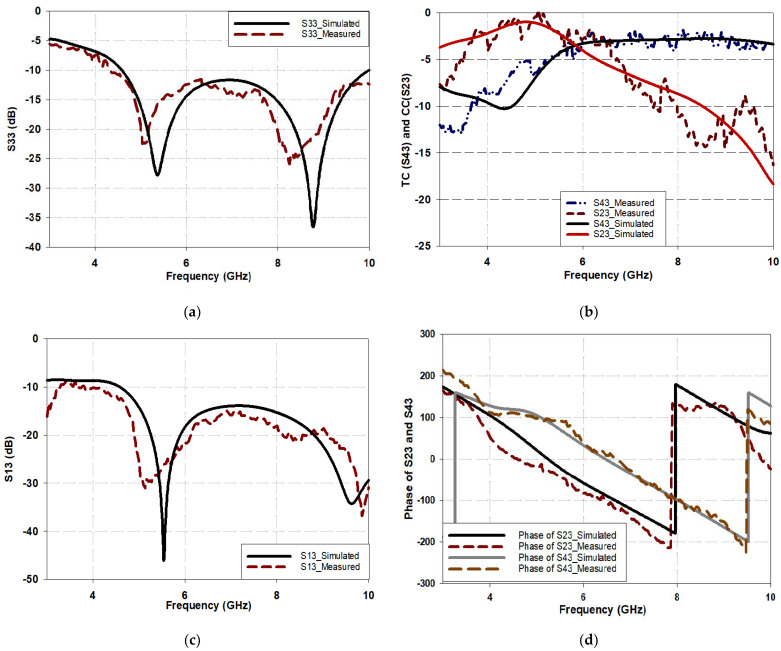
Performance of the proposed BLC when port 3 is excited: (**a**) S33, (**b**) S43 and S23 (**c**) S13, and (**d**) phase of S23 and S43.

Figure 12 presents the proposed BLC performance when port 3 is excited. From Figure 12a, it is evident that the −10 dB S_33_ bandwidth extends from 4.5 GHz to 10 GHz, which corresponds to a relative bandwidth of 75.8% compared to ~40% for the traditional branch line coupler based on the same design specifications and operating frequency of 6 GHz. It should be noted that a marginal difference of 1.9% occurs between the reflection coefficients’ bandwidths of S_11_ and S_33_. The transmission coefficient, S_23_, and the coupling coefficient, S_43_, for port 3 excitations are illustrated in Figure 12b. At the target band, i.e., at 6 GHz, the transmission and the coupling coefficients of the proposed BLC design are −3.9 dB, which is close to the ideal value of −3 dB. Notably, the transmission coefficient, S_43_, remain higher than −3.5 dB for the entire operating band ranging from 5.8 GHz to 10 GHz, and this behavior is consistent throughout this wide operating frequency range. However, the coupling coefficient, S_23_, gradually degrades with increasing frequency, and this is due to the use of vias as well as losses inherited from the lossy FR4 substrates, as the input port and coupled ports are located on opposite sides of different substrates. Despite this degradation, the power delivered to port 4 remains greater than −7.5 dB up to a frequency of 8 GHz. In Figure 12c, the isolation coefficient between input ports, S_13_, is depicted. It is evident that S_13_ remains less than –14 dB throughout the operating band. It can be concluded that the proposed miniaturized BLC has excellent performance in terms of scattering parameters (S-parameters) compared to a traditional BLC design. Additionally, based on the obtained results, it can also be confirmed that the ports are reciprocal and have the same S-parameters characteristics for all ports. This consolidates the principle that the proposed miniaturized BLC can be used as a unit cell for constructing a Butler matrix, which in turn has potential use in the development of phased array antenna systems.

Figure 12d illustrates the phase difference between output ports 2 and 4 for input excitations from port 3. At a frequency of 6 GHz, the phase difference between output ports for input from port 3 (∠S_43_ − ∠S_23_ = 94.8°) meets the requirements of a BLC coupler for good performance. However, a slight discrepancy between the simulated and measured phases is observed for input port 3. This difference may be attributed to fabrication tolerance and the lump solder for SMA feeders. Nevertheless, the phase difference error (PDE) at a design frequency of 6 GHz is 4.8° for input port 3 excitation.

Table 2 provides a comparison between the performance of the proposed BLC and those of previously published BLC designs. Most of the designs presented in Table 2 were focused on either improving the bandwidth or reducing the size of the BLC. The proposed work, however, achieves both bandwidth enhancement and size reduction, which is crucial for the design of a 5G system. Furthermore, when compared to the literature, the proposed miniaturized wide band BLC offers other advantages such as design simplicity, ease of fabrication, smaller phase difference errors, and equal power distribution among output ports at a design frequency of 6 GHz.

## 5. Conclusions

A novel quasi-twisted miniaturized wideband branch line coupler was introduced, which employs a Z-shaped meandered microstrip line with a slow wave structure on a double-layer substrate. The performance of the proposed design was evaluated, demonstrating agreement between the simulation and measurements and showing advantages over conventional microstrip branch line couplers in terms of size reduction and band improvement.

The novel quasi-twisted miniaturized wideband branch line coupler operates within the frequency band of 4.5 GHz to 10 GHz, offering a high relative bandwidth of up to 75.8% and reducing the size by 49.9% compared to conventional branch line couplers with a general bandwidth of 42.2%. A comparison between the proposed branch line coupler and the conventional design indicates that the former exhibits favorable scattering parameter specifications. Furthermore, the obtained results suggest that the ports of the branch line coupler demonstrate reciprocity, benefiting from consistent S-parameter characteristics across all ports. As a result, the proposed branch line coupler holds the potential to be integrated into a compact and wideband Butler matrix for future deployment in phased antenna array systems for potential use in 5G applications.

## Figures and Tables

**Table 1:** The design specifications of the suggested BLC.

Description	Notation	Dimension (λ_g_ at 6 GHz)	Description	Notation	Dimension (λ_g_ at 6 GHz)
Lengths of input arm (*P*_1_) and Isolated arm (*P*_3_)	*L_I_*	0.33	Coupled arm (*P*_4_) length	*L_c_*	0.359
All ports’ width	*W_I_*	0.054	Via radius	*V_R_*	0.027
Horizontal arm (port) length	*L_H_*	0.311	Substrates’ thickness	*H*_1_&*H*_2_	0.0286
Horizontal arm (port) Width	*W_H_*	0.086	Substrates’ Length	*L_S_*	0.645
Output arm (*P*_2_) length	*L_o_*	0.305	Substrates’ Width	*W_S_*	0.304
Horizontal arm (port) Width	*W_H_*	0.086	Open-circuited stub length	*L_ST_*	0.022
Open-circuited stub width	*W_ST_*	0.0134	Actual circuit area	*L_c_* × *W_c_*	0.359 × 0.23

**Table 2:** Comparison of proposed BLC performance with respect to published designs available in the literature.

Ref.	Operating Frequency (GHz)	Relative Bandwidth (%)	$\mathrm{Size}\;(\lambda_{g}^{2})\;$	S_11_ (dB)	S_21_ (dB)	S_41_ (dB)	S_31_ (dB)	PDE Error (Deg)
[4]	3.5	30.22	0.27 × 0.16	−27.47	−4.4	−3.1	−26.2	3.54
[5]	2.36	31.77	0.25 × 0.29	−25.5	−2.9	−3.2	−27.5	1
[7]	1.77	7.2	0.62 × 0.62	−35.9	−3.1	−3	−37.9	-
[8]	0.9	67.7	0.2 × 0.2	−19.89	−3.69	−3.67	−17.5	-
[9]	3	50.4	0.45 × 0.25	−21	−0.95	−10.5	−29	±10
[1]	3.5	40.2	0.55 × 0.65	−12	−3.19	−2.8	−15.2	−2
[11]	0.95	22.2	0.17 × 0.08	−30	2.3	−3.2	29	-
[12]	2.4	33.3	0.2 × 0.35	−20	12.5	−2.5	−20	±13
[13]	2	13 (S11 < −20)	0.3 × 0.3	−30	−3.5	−3.5	−27	±5
This Work	6	75.8	0.3 × 0.74	−14	−3.17	−3.17	−18.9	0.5

## Data Availability

Data are contained within the article.

## References

[1] Kiani S.H., Altaf A., Anjum M.R., Afridi S., Arain Z.A., Anwar S., Khan S., Alibakhshikenari M., Lalbakhsh A., Khan M.A. (2021). MIMO antenna system for modern 5G handheld devices with healthcare and high rate delivery. Sensors.

[2] Vallappil A.K., Rahim M.K.A., Khawaja B.A., Aminu-Baba M. (2020). Metamaterial based compact branch-line coupler with enhanced bandwidth for use in 5G applications. Appl. Comput. Electromagn. Soc. J. (ACES).

[3] Zhang J., Ge X., Li Q., Guizani M., Zhang Y. (2016). 5G millimeter-wave antenna array: Design and challenges. IEEE Wirel. Commun..

[4] Gomha S., El-Rabaie E.-S.M., Shalaby A.-A.T., Elkorany A.S. (2015). Design of new compact branch-line coupler using coupled line dual composite right/left-handed unit cells. J. Optoelectron. Adv. Mater..

[5] Abdulbari A.A., Rahim S.K.A., Abd Aziz M.Z.A., Tan K.G., Noordin N., Nor M. (2021). New design of wideband microstrip branch line coupler using T-shape and open stub for 5G application. Int. J. Electr. Comput. Eng..

[6] Edwards T.C., Steer M.B. (2016). Foundations for Microstrip Circuit Design.

[7] Alhalabi H., Issa H., Pistono E., Kaddour D., Podevin F., Baheti A., Abouchahine S., Ferrari P. (2018). Miniaturized branch-line coupler based on slow-wave microstrip lines. Int. J. Microw. Wirel. Technol..

[8] Chen W.L., Wang G.M. (2008). Design of novel miniaturized fractal-shaped branch-line couplers. Microw. Opt. Technol. Lett..

[9] Xu H.-X., Wang G.-M., Liang J.-G. (2011). Novel composite right-/left-handed transmission lines using fractal geometry and compact microwave devices application. Radio Sci..

[10] Xu H.-X., Wang G.-M., Chen X., Li T.-P. (2011). Broadband balun using fully artificial fractal-shaped composite right/left handed transmission line. IEEE Microw. Wirel. Compon. Lett..

[11] Gomha S., EL-Sayed M., Shalaby A.A.T., Ahmed S. (2014). Miniaturization of Branch-line couplers using open stubs and stepped impedance unit cells with meandering transmission lines. Circuits Syst. Int. J. (CSIJ).

[12] Kumar K.V.P., Alazemi A.J. (2021). A flexible miniaturized wideband branch-line coupler using shunt open-stubs and meandering technique. IEEE Access.

[13] Kumar M., Islam S.N., Sen G., Parui S.K., Das S. (2018). Design of miniaturized 10 dB wideband branch line coupler using dual feed and T-shape transmission lines. Radioengineering.

[14] Nie W., Xu K.-D., Zhou M., Xie L.-B., Yang X.-L. (2019). Compact narrow/wide band branch-line couplers with improved upper-stopband. AEU-Int. J. Electron. Commun..

[15] Siahkamari H., Jahanbakhshi M., Al-Anbagi H.N., Abdulhameed A.A., Pokorny M., Linhart R. (2022). Trapezoid-shaped resonators to design compact branch line coupler with harmonic suppression. AEU-Int. J. Electron. Commun..

[16] Wang X.-Z., Chen F.-C., Chu Q.-X. (2023). A Compact Broadband 4 × 4 Butler Matrix with 360° Continuous Progressive Phase Shift. IEEE Trans. Microw. Theory Tech..

[17] Mextorf H., Schernus W. (2023). A Novel Branch-Line Coupling Topology. IEEE Trans. Microw. Theory Tech..

[18] Roshani S., Yahya S.I., Roshani S., Rostami M. (2022). Design and fabrication of a compact branch-line coupler using resonators with wide harmonics suppression band. Electronics.

[19] Majidifar S., Hayati M. (2017). New approach to design a compact triband bandpass filter using a multilayer structure. Turk. J. Electr. Eng. Comput. Sci..

[20] Pozar D.M. (2011). Microwave Engineering.

[21] Tian H., Gao J., Su M., Wu Y., Liu Y. (2015). A novel 3D two-ways folded microstrip line and its application in super-miniaturized microwave wireless components. J. Electromagn. Waves Appl..

[22] LaMeres B.J. (2000). Characterization of a Printed Circuit Board via.

[23] Rawat K., Ghannouchi F. Design of reduced size power divider for lower RF band using periodically loaded slow wave structure. Proceedings of the 2009 IEEE MTT-S International Microwave Symposium Digest.

[24] Eccleston K.W., Ong S.H. (2003). Compact planar microstripline branch-line and rat-race couplers. IEEE Trans. Microw. Theory Tech..

[25] Mayer B., Knochel R. Branchline-couplers with improved design flexibility and broad bandwidth. Proceedings of the IEEE International Digest on Microwave Symposium.

